# Supplementary material for: Impact of BRCA Mutation Status on Tumor Infiltrating Lymphocytes (TILs), Response to Treatment, and Prognosis in Breast Cancer Patients Treated with Neoadjuvant Chemotherapy
Source: Cancers (Basel). 2020 Dec 8;12(12):3681. doi: 10.3390/cancers12123681 (PMC7764707; doi:10.3390/cancers12123681)
Supplement: Supplementary file 1 [file cancers-12-03681-s001.pdf]

Supplementary materials

Impact of *BRCA* Mutation Status on Tumor Infiltrating Lymphocytes (TILs), Response to Treatment, and Prognosis in Breast Cancer Patients Treated with Neoadjuvant Chemotherapy

Beatriz Grandal [1,2,†], Clémence Evrevin [1,†], Enora Laas [1], Isabelle Jardin [1], Sonia Rozette [1], Lucie Laot [1], Elise Dumas [2], Florence Coussy [3], Jean-Yves Pierga [3], Etienne Brain [4], Claire Saule [5], Dominique Stoppa-Lyonnet [5], Sophie Frank [5], Claire Sénéchal [6], Marick Lae [7,8], Diane De Croze [7], Guillaume Bataillon [9], Julien Guerin [10], Fabien Reyal [1,2,*,‡] and Anne-Sophie Hamy [2,‡]

1 Department of Surgery, Institut Curie, University Paris, 75005 Paris, France; beatriz.grandalrejo@curie.fr (B.G.); clemence.evrevin@gmail.com (C.E.); enora.laas@curie.fr (E.L.); isabelle_jardin@hotmail.fr (I.J.); sonia_rozette@hotmail.fr (S.R.); lucielaot@yahoo.fr (L.L.)
2 Residual Tumor & Response to Treatment Laboratory, RT2Lab, Translational Research Department, INSERM, U932 Immunity and Cancer, Institut Curie, 26 rue d'Ulm, 75005 Paris, France; elise.dumas@curie.fr (E.D.); anne-sophie.hamy-petit@curie.fr (A.-S.H.)
3 Department of Oncology, Institut Curie, 26 rue d'Ulm, 75005 Paris, France; florence.coussy@curie.fr (F.C.); jean-yves.pierga@curie.fr (J.-Y.P.)
4 Department of Oncology, Centre René Huguenin, Institut Curie, 35 rue Dailly, 92210 St Cloud, France; etienne.brain@curie.fr
5 Department of Genetics, Institut Curie, 26 rue d'Ulm, 75005 Paris, France; claire.saule@curie.fr (C.S.); Dominique.StoppaLyonnet@curie.fr (D.S.-L.); sophie.frank@curie.fr (S.F.)
6 Department of Genetics, Institut Bergonié, 229 Cours de l'Argonne, 33000 Bordeaux, France; c.senechal-davin@bordeaux.unicancer.fr
7 Department of Pathology, Centre René Huguenin, Institut Curie, 35 rue Dailly, 92210 St Cloud, France; marick.lae@curie.fr (M.L.); diane.decroze@curie.fr (D.D.C.)
8 Department of Pathology, Centre Henri Becquerel, INSERM U1245, UNIROUEN, University of Normandie, 76038, Rouen, France
9 Department of Pathology, Institut Curie, 26 rue d'Ulm, 75005 Paris, France; guillaume.bataillon@curie.fr
10 Data Office, Institut Curie, 25 rue d'Ulm, 75005 Paris, France; julien.guerin@curie.fr
* Correspondence: fabien.reyal@curie.fr; Tel.: +33-144324660; Fax: +33-153104037
† These authors equally contributed to this work.
‡ These authors equally contributed to this work.

Received: 2 November 2020; Accepted: 3 December 2020; Published: 7 December 2020

SUPPLEMENTARY MATERIAL

Patients and treatments

Patients

In total, patients with T1-3NxM0 invasive breast cancer (BC) (NEOREP Cohort, CNIL declaration number 1547270) treated at Institut Curie (Paris and Saint Cloud) between 2002 and 2012 were included in this study. We included unilateral, non-recurrent, non-inflammatory, non-metastatic tumors, excluding T4 tumors. NAC regimens changed over time (anthracycline-based regimen or sequential anthracycline-taxane regimen) with trastuzumab used in an adjuvant and/or neoadjuvant setting since 2005 for *HER2*-positive tumors. All patients underwent radiotherapy. Endocrine therapy (tamoxifen or aromatase inhibitor) was prescribed when indicated. This study was approved by the Breast Cancer Study Group of Institut Curie.

Treatments

NAC regimens changed over time (anthracycline-based regimen or sequential anthracycline-taxane regimen), with trastuzumab used in an adjuvant and/or neoadjuvant setting for HER2-positive tumors since the middle of the past decade. Trastuzumab treatments changer over time due to a change of marketing authorization during the study period. Adjuvant hormone therapy (tamoxifen, aromatase inhibitor, or GnRH agonist) was prescribed when indicated. Surgery (breast-conserving or mastectomy) was performed 4-6 weeks after NAC. Every patient received adjuvant radiotherapy. Adjuvant chemotherapy (ADJ) was decided after multidisciplinary consultation meeting considering patient characteristic, prognosis factor and response to NAC (residual disease and/or node involvement). Patient follow-up after treatment was of every 4 months during the first 2 years, then every 6 months for 3 years, and once a year starting from the 5th year. Follow-up consisted of clinical examination associated to mammography and mammary ultrasound once a year, with annual Magnetic resonance imaging (RMI) in *BRCA*-carriers.

Tumor samples and pathological review

ER, PR, HER2 status and BC subtype

Cases were considered to be estrogen receptor (ER)-positive or progesterone receptor (PR)-positive if at least 10% of the tumor cells expressed estrogen and/or progesterone receptors (ER/PR). *HER2* expression was determined by immunohistochemistry, with scoring according to the American Society of Clinical Oncology (ASCO)/College of American Pathologists (CAP) guidelines [1]. Scores of 3+ were reported as positive, and scores of 1+/0 as negative. Tumors with scores of 2+ were further tested by fluorescence *in situ* hybridization (FISH). For *HER2* gene amplification, we evaluated a mean of 40 tumor cells per sample and calculated the mean *HER2* signal per nucleus. A *HER2*/CEN17 ratio ≥ 2 was considered positive, and a ratio < 2 was considered negative [1].

Other pathological parameters

Histological grade was determined as described by Elston Ellis. Mitotic cells were counted on 10 high-power fields (HPF) (x40 objective; field diameter = 0.62 mm) and cutoffs of <11, 12–22 and >22 mitoses were used to define low, intermediate and high mitotic indices, respectively, according to the international recommendations[2]. Due to significant differences in distribution before and after NAC, invasive tumor cellularity was binned according to the median value (pre-NAC: 60%; post-NAC: 30%).

BRCA status

Since 2002, patient referral for genetic counseling depends on individual or family criteria. These criteria are based on the probability of identifying a genetic predisposition in the family of at least 10% (in particular a germline *BRCA1* or *BRCA 2* pathogenic variant). The individual criteria are: early age at diagnosis (under 40) or bilateral breast cancer: synchronous or metachronous (with the first breast cancer before age 50), or specific phenotype (triple negative cancer before age 51).The family criteria are: 3 cases of breast cancer in the same branch of heredity, or 2 cases of breast cancer including 1 under 45-50, of breast or ovarian cancer, or 2 cases of breast cancer including 1 male. The 2 cases are women relatives of the first degree (or second degree if paternal transmission).

TILs levels

Infiltrates were scored on a continuous scale, as the mean percentage of the stromal area occupied by mononuclear cells. After NAC, we assessed TIL levels within the borders of the residual tumor bed, as defined by the RCB index[3]. Nothing is known about the clinical, biological and prognostic significance of TILs in the area of regression in cases of pathological response, but the TILs international working group recently called for their evaluation for research purposes. In cases of pCR, the scar area was measured on macroscopic examination. The scar appeared as a white area in the breast parenchyma corresponding to the tumor bed modified by NAC. It was characterized by

the presence of histiocytes, lymphocytes, macrophages, fibrosis and elastosis. The whole fibro-inflammatory scar was evaluated on HE sections (size in mm and stromal TIL level evaluation).

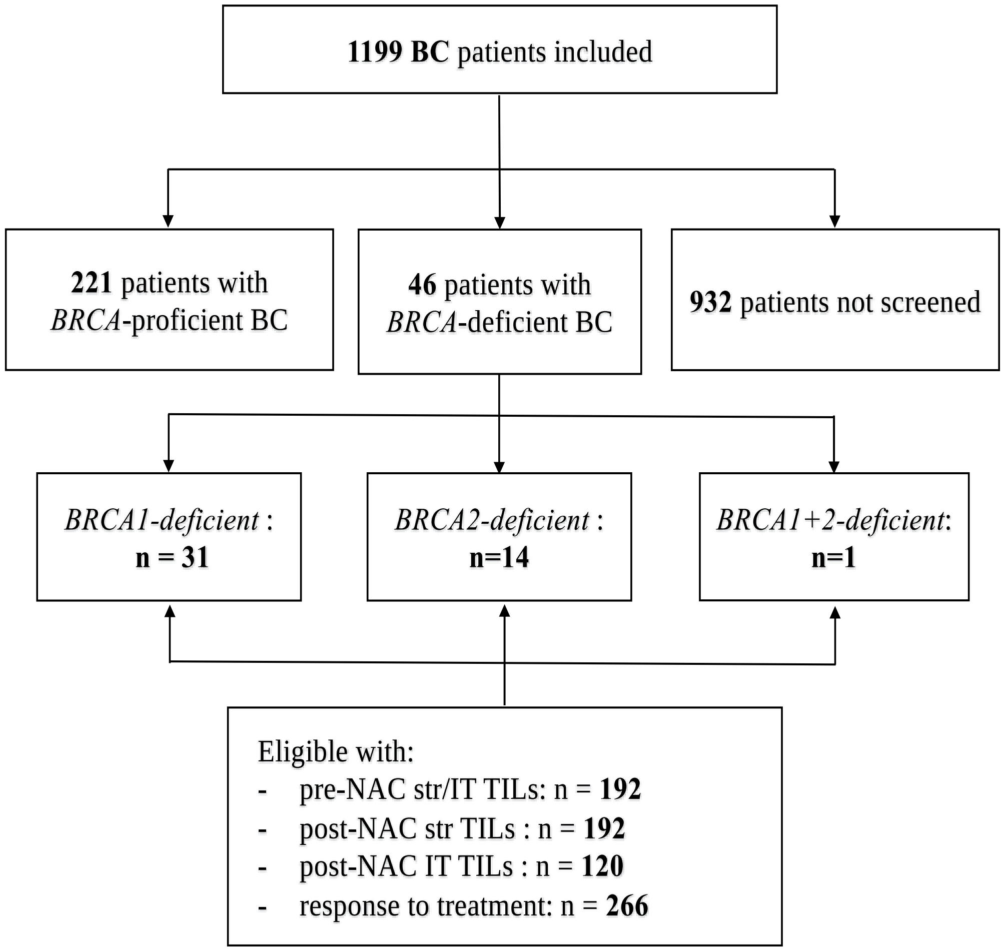

Figure S1. Study flow diagram of included patients and tumors samples available.

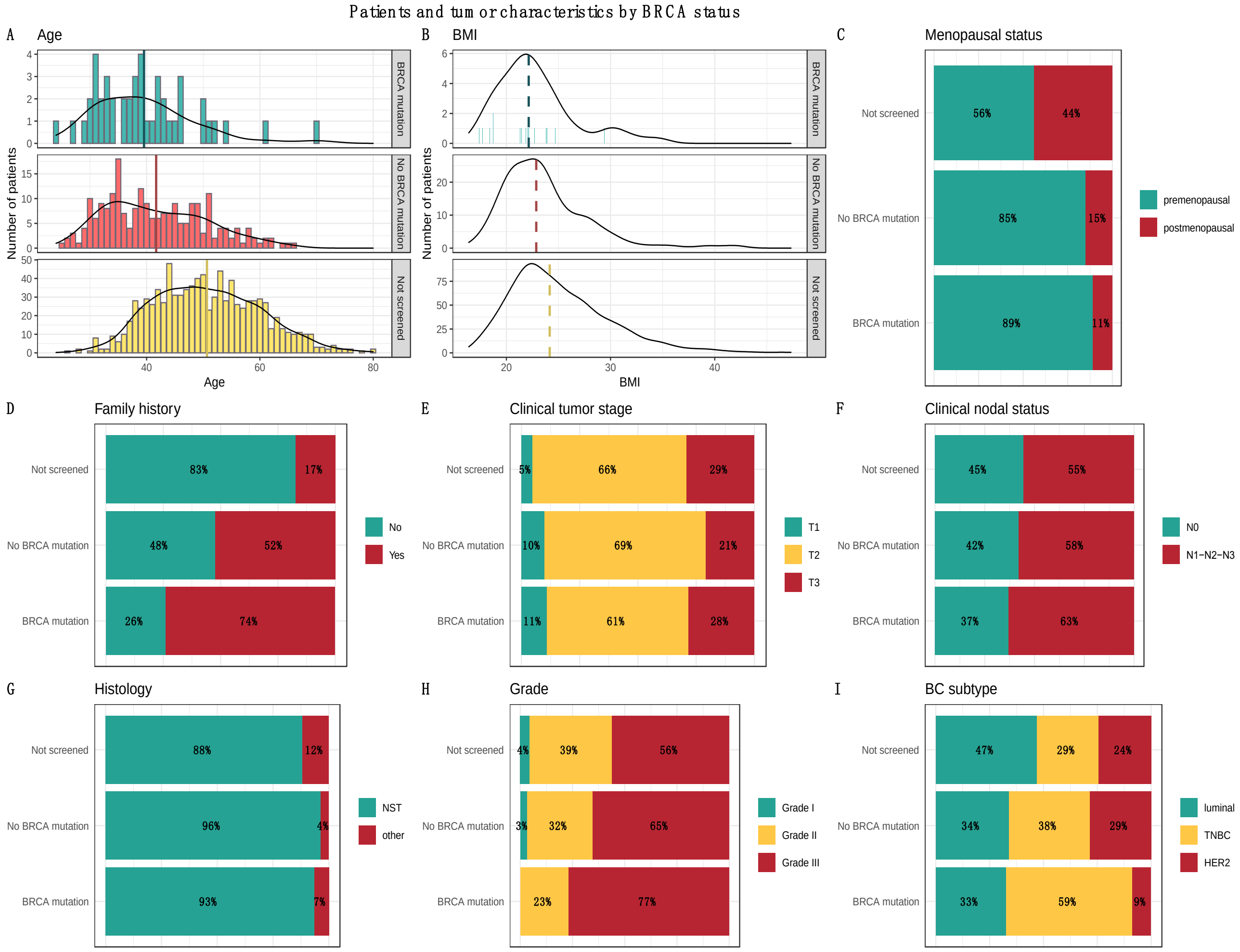

Figure S2. Patients´ and tumors ´characteristics by BRCA status. (All(n=1199), BRCA mutation (n=36), BRCA wild-type(n=156), not screened (n=1007). **A,** Age (kernel density plot). **B,** BMI (kernel density plot). **C,** Menopausal status (barplot). **D,** Family history (barplot). **E,** Clinical tumor stage (barplot). **F,** Clinical nodal status (barplot). **G,** Histology (barplot). **H**, Grade (barplot). **I,** BC subtype (barplot).

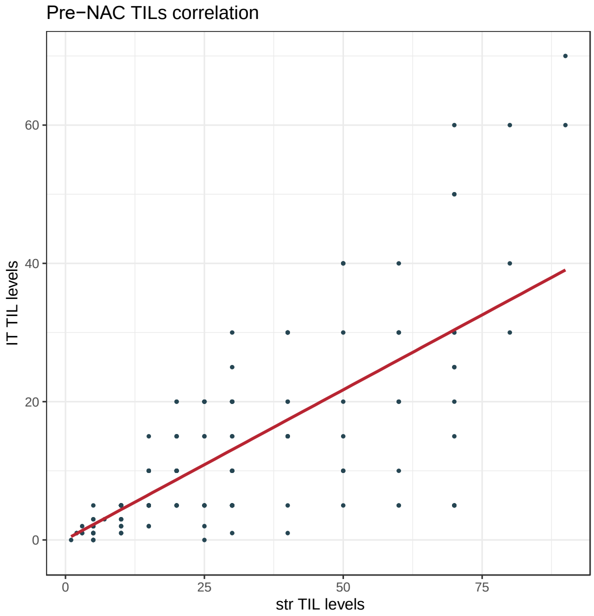

Figure S3. Variation of pre-NAC str TIL levels according to the pre-NAC IT TIL levels (scatterplot) (str TILs (n=192), IT TILs (n=192)).

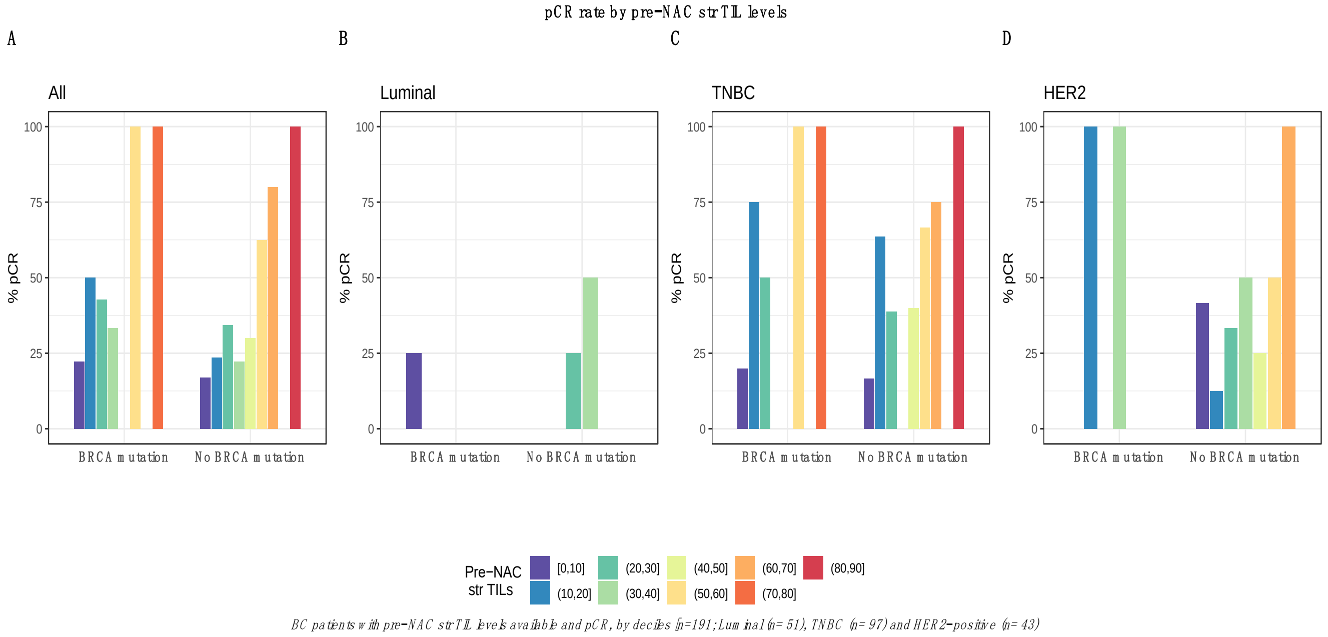

Figure S4. pCR rate by pre-NAC str TIL levels by BRCA status (TILs were binned by increments of 10%). **A**, whole population (n=191, BRCA mutation (n=36), BRCA wild-type(n=155)). **B,** luminal tumors (n=51, BRCA mutation(n=8), BRCA wild-type(n=43))**. C,** TNBC (n=97), BRCA mutation(n=24), BRCA wild-type(n=73)). **D,** HER2-positive BC (n=43, BRCA mutation(n=4), BRCA wild-type(n=39)).

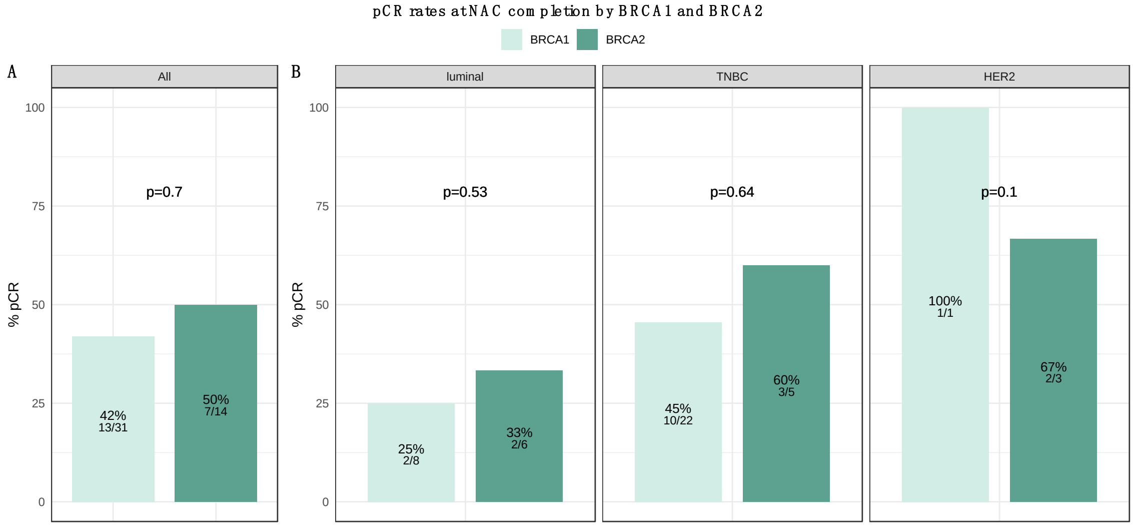

Figure S5. Barplot of associations between response to treatment and BRCA pathogenic mutations treated with NAC. **A**, among the whole population (All(n=45), BRCA1 (n=31), BRCA2 (n=14)). **B,** by BC subtype (Luminal (n=14), BRCA1 (n=8), BRCA2 (n=6)); TNBC (n=27), BRCA1 (n=22), BRCA2 (n=5)); HER2 (n=4), BRCA1 (n=1), BRCA2 (n=3)).

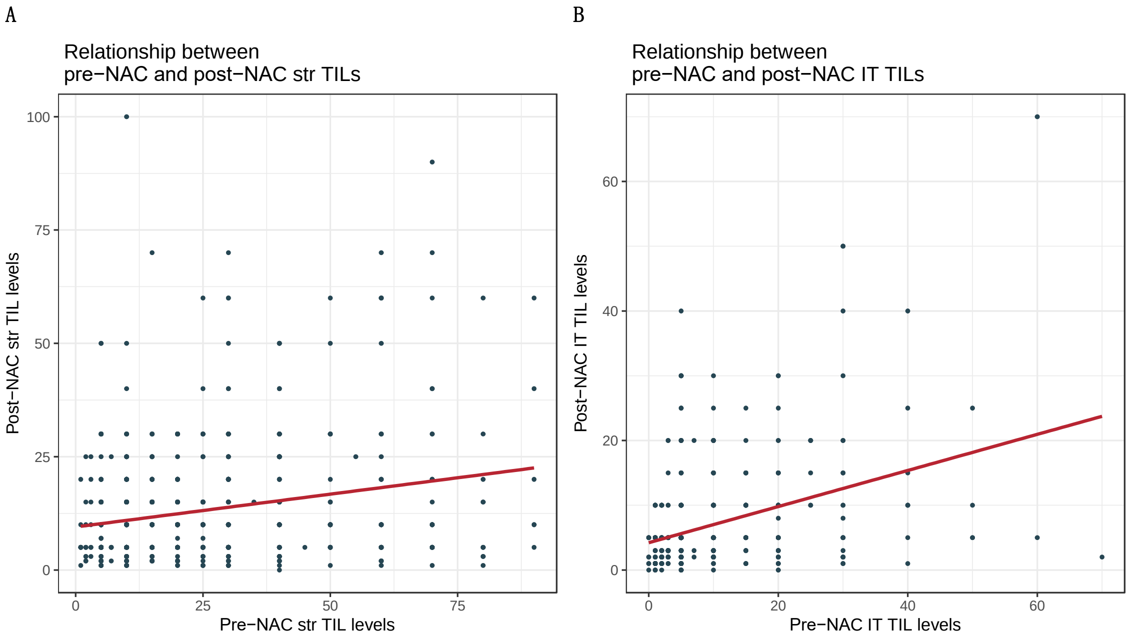

Figure S6. TILs correlation between pre and post-NAC. **A,** Variation of post-NAC str TIL levels according to the pre-NAC str TIL levels (scatterplot) (pre-NAC str TILs (n=192), post-NAC str TILs (n=192)). **B,** Variation of post-NAC IT TIL levels according to the pre-NAC IT TIL levels (scatterplot) (pre-NAC IT TILs (n=192), post-NAC IT TILs (n=120)).

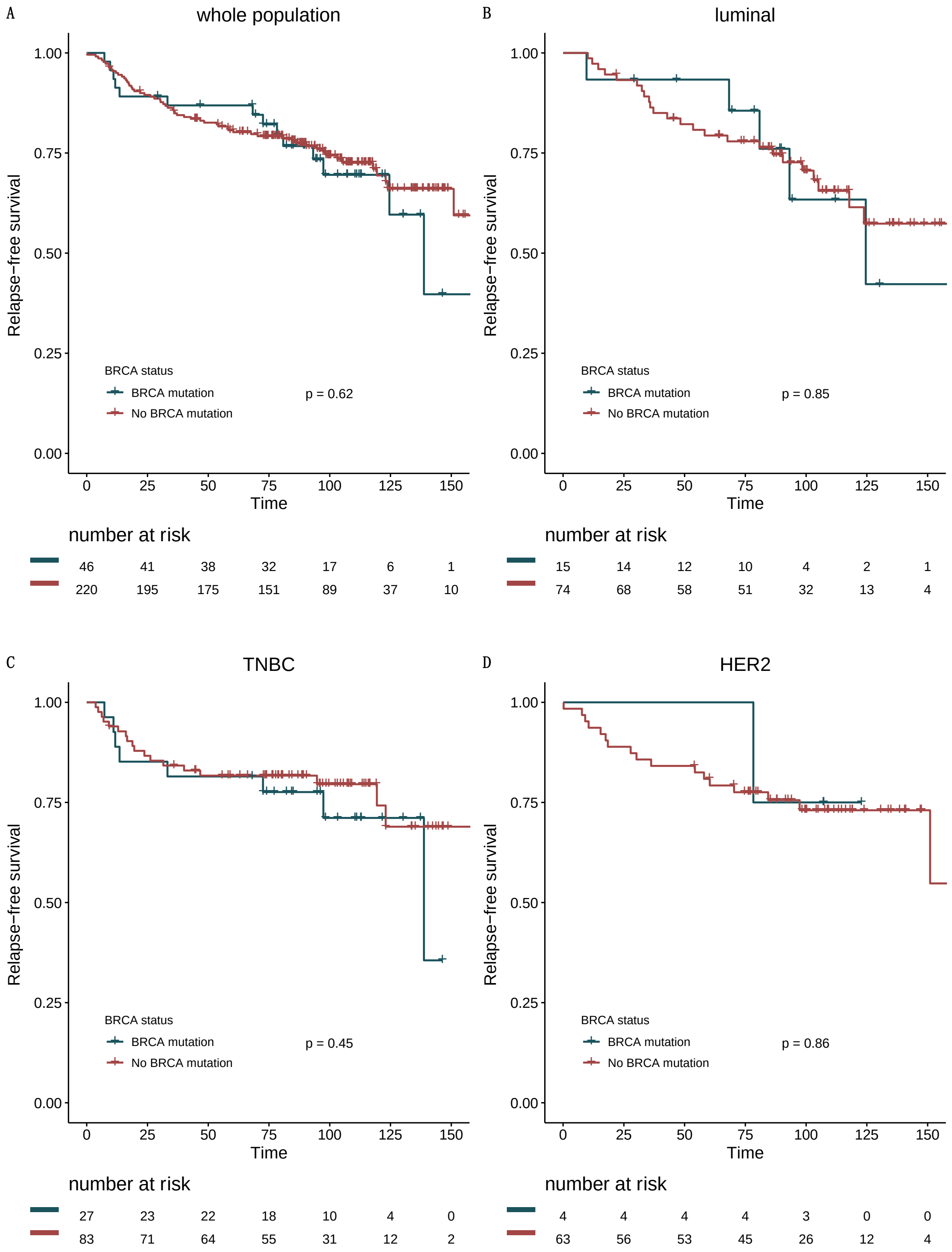

Figure S7. Relapse free survival curves according BRCA status. **A**, whole population (n=267, BRCA mutation (n=46), BRCA wild-type(n=220)). **B**, luminal tumors (n=89, BRCA mutation(n=15), BRCA wild-type(n=74)). **C**, TNBC (n=110, BRCA mutation(n=27), BRCA wild-type(n=83)). **D,** HER2-positive BC (n=67), BRCA mutation(n=4), BRCA wild-type(n=63)).

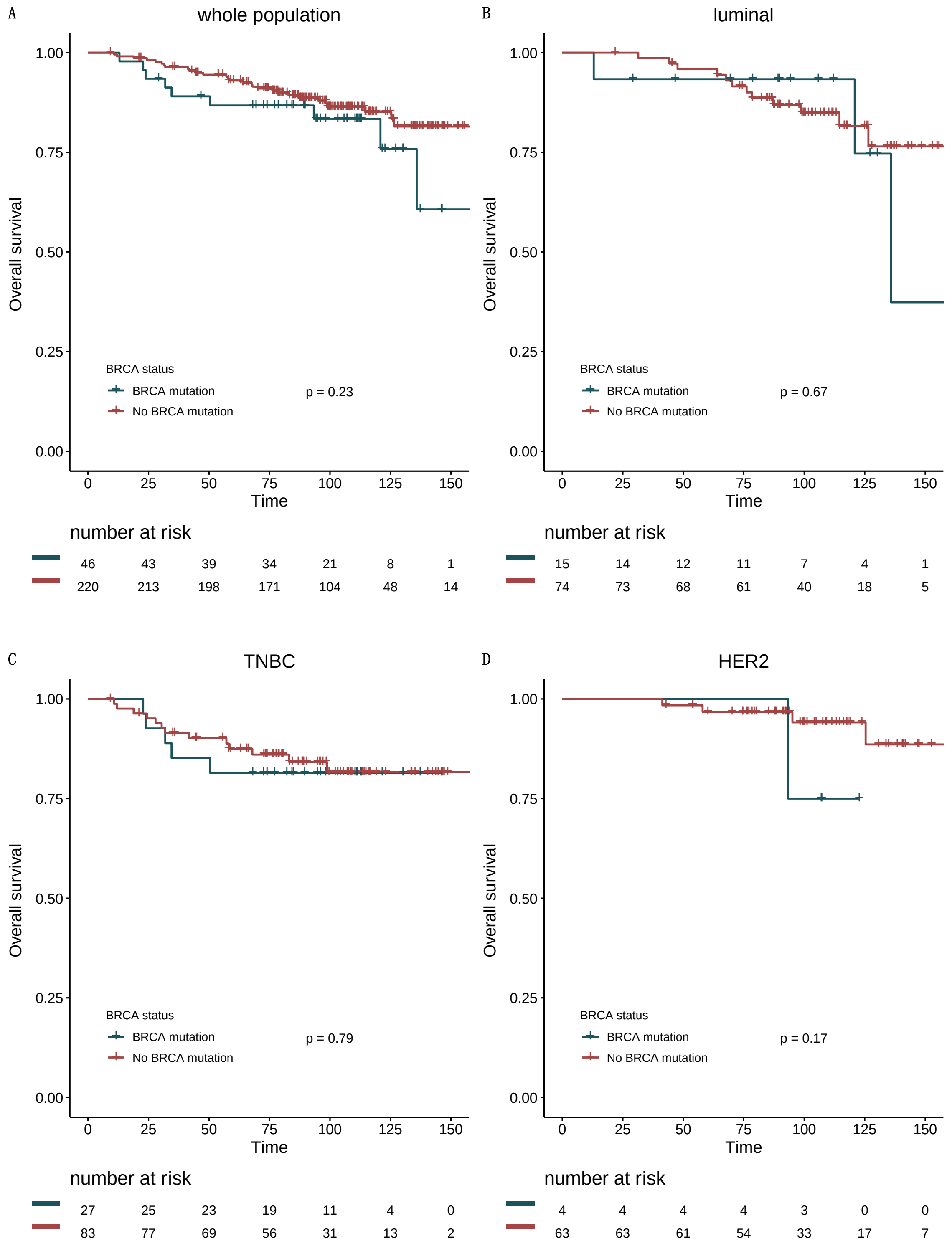

Figure S8. Overall survival curves according BRCA status. **A**, whole population (n=267, BRCA mutation (n=46), BRCA wild-type(n=220)). **B**, luminal tumors (n=89, BRCA mutation(n=15), BRCA wild-type(n=74)). **C**, TNBC (n=110, BRCA mutation(n=27), BRCA wild-type(n=83)). **D,** HER2-positive BC (n=67), BRCA mutation(n=4), BRCA wild-type(n=63)).

Table S1. BRCA screened patients´characteristics among by BRCA status.

Characteristics	Class	Overall	BRCA mutation	BRCA wild-type	*p*
**n=**		**267 (100%)**	**46(17%)**	**221(83%)**	
**Age (mean)**		41.31	39.5	41.7	0.15
**Menopausal status**	pre	228 (85.7%)	41 (89.1%)	187 (85.0%)	0.62
	post	38 (14.3%)	5 (10.9%)	33 (15.0%)	
**BMI (mean)**		23.50	22.8	23.6	0.24
**BMI class**	[15,19]	23 (8.6%)	6 (13.3%)	17 (7.7%)	0.248
	(19,25]	178 (66.9%)	31 (68.9%)	147 (66.5%)	
	(25,30]	47 (17.7%)	4 (8.9%)	43 (19.5%)	
	(30,50]	18 (6.8%)	4 (8.9%)	14 (6.3%)	
**Family history of BC**	no	116 (43.9%)	12 (26.1%)	104 (47.7%)	**0.01**
	yes	148 (56.1%)	34 (73.9%)	114 (52.3%)	
**Clinical tumor size**	T1	27 (10.1%)	5 (10.9%)	22 (10.0%)	0.50
	T2	181 (67.8%)	28 (60.9%)	153 (69.2%)	
	T3	59 (22.1%)	13 (28.3%)	46 (20.8%)	
**Clinical nodal status**	N0	110 (41.2%)	17 (37.0%)	93 (42.1%)	0.63
	N1-N2-N3	157 (58.8%)	29 (63.0 %)	128 (57.9%)	
**Histology**	NST	256 (95.9%)	43 (93.5%)	213 (96.4%)	NaN
	others	11 (4.1%)	3 (6.5%)	8 (3.6%)	
**Grade**	I-II	86 (32.8%)	10 (23.3%)	76 (34.7%)	0.24
	III	176 (67.2%)	33 (76.7%)	143 (65.3%)	
**Mitotic Index (mean)**		26.57	30.8	25.6	0.24
**Subtype**	luminal	90 (33.7%)	15 (32.6%)	75 (33.9%)	**<0.01**
	TNBC	110 (41.2%)	27 (58.7%)	83 (37.6%)	
	HER2	67 (25.1%)	4 (8.7%)	63 (28.5%)	
**str TILs (mean)**		20.0 [10.0-40.0]	20.0 [13.8-40.0]	20.0 [10.0-40.0]	0,78
**IT TILs (mean)**		5.0 [5.0-15.0]	5.0 [5.0-11.3]	7.5 [5.0-20.0]	0,72
**NAC Regimen**	AC	29 (10.9%)	4 (8.7%)	25 (11.4%)	0.49
	AC-Taxanes	221 (83.1%)	41 (89.1%)	180 (81.8%)	
	Taxanes	7 (2.6%)	1 (2.2%)	6 (2.7%)	
	Others	9 (3.4%)	0 (0.0%)	9 (4.1%)	
**pCR class**	No pCR	182 (68.4%)	25 (54.3%)	157 (71.4%)	**0,04**
	pCR	84 (31.6%)	21 (45.7%)	63 (28.6%)	
**Nodal involvment**	0	176 (66.2%)	35 (76.1%)	141 (64.1%)	0,16
	1-3	64 (24.1%)	6 (13.0%)	58 (26.4%)	
	≥4	26 (9.8%)	5 (10.9%)	21 (9.5%)	
**str TILs (mean)**		10.0 [5.0-15.0]	15.0 [5.0-20.0]	10.0 [5.0-15.0]	0,14
**IT TILs (mean)**		5.0 [2.0-10.0]	5.0 [4.3-10.0]	5.0 [2.0-10.0]	0,27

Missing data: Menopausal status, *n* = 1; BMI (continuous), n=1; BMI class, n=1; Family history, *n* = 3; Grade, *n* = 5; Mitotic index, *n* = 77; Pre-NAC str TILs, *n* = 75; Pre-NAC IT TILs, *n* = 75; NAC regimen, *n* = 1;pCR status, *n* = 1; Post-NAC Nodal involvment, *n* = 1; Post-NAC str TILs, *n* = 75; Post-NAC IT TILS, *n* = 147.

Abbreviations: NAC=neoadjuvant chemotherapy ; BMI=body mass index; NST= no special type ; TNBC= triple negative breast cancer ; str TILs= stromal tumor-infiltrating lymphocytes ; IT TILs= intratumoral-infiltrating lymphocytes; AC=anthracyclines; pCR=Pathologic complete response.

The "n" denotes the number of patients. In case of categorical variables, percentages are expressed between brackets. In case of continuous variables, mean value is reported. In case of nonnormal continuous variables, median value is reported, with interquartile range between brackets.

Table S2. Patients´characteristics in each tumor subtype and by BRCA status.

Characteristics	Class	Luminal BRCA mutation	Luminal BRCA wild-type	Luminal *p*	TNBC BRCA mutation	TNBC BRCA wild-type	TNBC *p*	*HER2* BRCA mutation	*HER2* BRCA wild-type	*HER2* *p*
**n=**		**15 (16.7%)**	**75 (83.3%)**		**27 (24.5%)**	**83 (75.5%)**		**4 (6.0%)**	**63 (94.0%)**	
**Age (mean)**		38.5	42.3	0,13	39.6	42.2	0,19	43.5	40.2	0,50
**Menopausal status**	pre	14 (93.3%)	64 (86.5%)	0,76	24 (88.9%)	72 (86.7%)	1,00	3 (75.0%)	51 (81.0%)	1,00
	post	1 (6.7%)	10 (13.5%)		3 (11.1%)	11 (13.3%)		1 (25.0%)	12 (19.0%)	
**BMI (mean)**		22.2	23.5	0,32	23.5	23.3	0,74	20.4	24.3 (4.4%)	0,09
**BMI class**	[15,19]	4 (28.6%)	7 (9.3%)	0.204	1 (3.7%)	7 (8.4%)	0.251	1 (25.0%)	3 (4.8%)	0.298
	(19,25]	8 (57.1%)	49 (65.3%)		20 (74.1%)	55 (66.3%)		3 (75.0%)	43 (68.3%)	
	(25,30]	1 (7.1%)	14 (18.7%)		3 (11.1%)	18 (21.7%)		0 (0.0%)	11 (17.5%)	
	(30,50]	1 (7.1%)	5 (6.7%)		3 (11.1%)	3 (3.6%)		0 (0.0%)	6 (9.5%)	
**Family history**	no	2 (13.3%)	26 (35.1%)	0,18	10 (37.0%)	46 (56.1%)	0,13	0 (0.0%)	32 (51.6%)	0,14
	yes	13 (86.7%)	48 (64.9%)		17 (63.0%)	36 (43.9%)		4 (100.0%)	30 (48.4%)	
**Clinical tumor size**	T1	1 (6.7%)	5 (6.7%)	0,99	2 (7.4%)	11 (13.3%)	0,28	2 (50.0%)	6 (9.5%)	**0,04**
	T2	11 (73.3%)	54 (72.0%)		16 (59.3%)	56 (67.5%)		1 (25.0%)	43 (68.3%)	
	T3	3 (20.0%)	16 (21.3%)		9 (33.3%)	16 (19.3%)		1 (25.0%)	14 (22.2%)	
**Clinical nodal status**	N0	5 (33.3%)	35 (46.7%)	0,51	10 (37.0%)	34 (41.0%)	0,89	2 (50.0%)	24 (38.1%)	1,00
	N1-N2-N3	10 (66.7%)	40 (53.3%)		17 (63.0%)	49 (59.0%)		2 (50.0%)	39 (61.9%)	
**Histology**	NST	13 (86.7%)	72 (96.0%)	NaN	26 (96.3%)	78 (94.0%)	NaN	4 (100.0%)	63 (100.0%)	NaN
	others	2 (13.3%)	3 (4.0%)		1 (3.7%)	5 (6.0%)		0 (0.0%)	0 (0.0%)	
**Grade**	I-II	6 (42.9%)	42 (56.0%)	0,55	2 (8.0%)	10 (12.2%)	0,53	2 (50.0%)	24 (38.7%)	NaN
	III	8 (57.1%)	33 (44.0%)		23 (92.0%)	72 (87.8%)		2 (50.0%)	38 (61.3%)	
**Mitotic Index (mean)**		23.0	18.5	0,56	36.3	31.8	0,46	13.0	21.5	0,45
**str TILs (mean)**		12.5 [8.8-15.0]	15.0 [10.0-25.0]	0,17	27.5 [15.0-60.0]	30.0 [15.0-50.0]	0,71	22.5 [20.0-28.8]	20.0 [10.0-35.0]	0,66
**IT TILs (mean)**		5.0 [4.3-6.3]	10.0 [5.0-15.0]	0,07	5.0 [5.0-15.0]	5.0 [2.0-15.0]	0,37	15.0 [10.0-20.0]	15.0 [5.0-20.0]	0,77
**NAC Regimen**	AC	3 (20.0%)	13 (17.6%)	0,83	1 (3.7%)	9 (10.8%)	NaN	0 (0.0%)	3 (4.8%)	0,77
	AC-Taxanes	11 (73.3%)	58 (78.4%)		26 (96.3%)	73 (88.0%)		4 (100.0%)	49 (77.8%)	
	Taxanes	1 (6.7%)	2 (2.7%)		0 (0.0%)	0 (0.0%)		0 (0.0%)	4 (6.3%)	

	Others	0 (0.0%)	1 (1.4%)		0 (0.0%)	1 (1.2%)		0 (0.0%)	7 (11.1%)	
**pCR class**	No pCR	10 (66.7%)	70 (94.6%)	**<0,01**	14 (51.9%)	47 (56.6%)	0,83	1 (25.0%)	40 (63.5%)	0,32
	pCR	5 (33.3%)	4 (5.4%)		13 (48.1%)	36 (43.4%)		3 (75.0%)	23 (36.5%)	
**Nodal involvment**	0	8 (53.3%)	30 (40.5%)	0,23	23 (85.2%)	65 (78.3%)	0,74	4 (100.0%)	46 (73.0%)	0,49
	1-3	3 (20.0%)	32 (43.2%)		3 (11.1%)	13 (15.7%)		0 (0.0%)	13 (20.6%)	
	≥4	4 (26.7%)	12 (16.2%)		1 (3.7%)	5 (6.0%)		0 (0.0%)	4 (6.3%)	
**str TILs (mean)**		15.0 [13.8-20.0]	10.0 [5.0-10.0]	**<0,01**	10.0 [5.0-20.0]	10.0 [5.0-25.0]	0,82	10.0 [4.3-15.0]	10.0 [5.0-12.5]	0,95
**IT TILs (mean)**		10.0 [5.0-12.5]	5.0 [3.0-5.0]	**0,02**	5.0 [1.8-6.3]	5.0 [3.0-10.0]	0,54	5.0 [5.0-5.0]	5.0 [2.0-5.0]	0,68

Missing data: Menopausal status, n=1; BMI (continuous), *n* = 1; BMI class, *n* = 1; Family history, n=3; Grade, *n* = 5; Mitotic index, *n* = 77; Pre-NAC str TILs, *n* = 75; Pre-NAC IT TILs, *n* = 75; NAC regimen, *n* = 1;pCR status, *n* = 1; Post-NAC Nodal involvment, *n* = 1; Post-NAC str TILs, *n* = 75; Post-NAC IT TILS, *n* = 147. *Abbreviations:* NAC=neoadjuvant chemotherapy ; BMI=body mass index; NST= no special type ; TNBC= triple negative breast cancer ; str TILs= stromal tumor-infiltrating lymphocytes ; IT TILs= intratumoral-infiltrating lymphocytes; AC=anthracyclines; pCR=Pathologic complete response.

Table S3. Association of BRCA status with pCR after univariate and multivariate analysis in the whole population.

					Univariate				Multivariate		
**Variable**	**Class**	**Nb total**	**Nb in model**	**Events**	**HR**	**CI**	**RCH**	***p***	**HR**	**CI**	***p***
**Pre-NAC parameters**											
**Menopausal status**	pre	227	227	70	1		30.8 %				
	post	38	38	14	1.31	[0.63 - 2.65]	36.8 %	0,46			
**BMI class**	≤19	23	23	8	1		34.8 %				
	19-25	177	177	56	0.87	[0.36 - 2.27]	31.6 %	0,76			
	25-30	47	47	17	1.06	[0.38 - 3.11]	36.2 %	0,90			
	>30	18	18	3	0.37	[0.07 - 1.58]	16.7 %	0,20			
**BRCA status**	BRCA mutation	46	46	21	1		45.7 %				
	BRCA wild-type	220	220	63	0.48	[0.25 - 0.92]	28.6 %	**0,03**			
**Tumor size**	T1	27	27	15	1		55.6 %				
	T2	181	181	57	0.37	[0.16 - 0.83]	31.5 %	**0,02**	0.51	[0.17 - 1.49]	0.22
	T3	58	58	12	0.21	[0.08 - 0.55]	20.7 %	**<0.01**	0.26	[0.07 - 0.89]	**0.03**
**Clinical nodal status**	N0	110	110	33	1		30%				
	N1	145	145	48	1.15	[0.68 - 1.98]	33.1 %	0,61			
	N2	8	8	2	0.78	[0.11 - 3.58]	25%	0,77			
	N3	3	3	1	1.17	[0.05 - 12.59]	33.3 %	0,91			
**Grade**	I	7	7	1	1		14.3 %				
	II	78	78	17	1.67	[0.26 - 32.72]	21.8 %	0,65			
	III	176	176	66	3.6	[0.6 - 68.78]	37.5 %	0.24			
**Mitotic index**	≤22	101	101	29	1		28.7 %				
	>22	88	88	34	1.56	[0.85 - 2.89]	38.6 %	0.15	0.89	[0.42 - 1.83]	0.74
**BC subtype**	luminal	89	89	9	1		10.1 %				
	TNBC	110	110	49	7.14	[3.39 - 16.57]	44.5 %	**<0.01**	8.6	[2.76 - 33.41]	**<0.01**
	HER2	67	67	26	5.64	[2.5 - 13.78]	38.8 %	**<0.01**	6.31	[1.99 - 24.48]	**<0.01**
**str TILs (%)**				84	1.03	[1.02 - 1.05]		**<0.01**	1.01	[0.99 - 1.04]	0.33
**IT TILs (%)**				84	1.04	[1.02 - 1.07]		**<0.01**	1.03	[0.98 - 1.07]	0.24
**NAC regimen**	AC	29	29	7	1		24.1 %				
	AC-Taxanes	221	221	72	1.52	[0.65 - 3.99]	32.6 %	0,36			
	Taxanes	7	7	3	2.36	[0.39 - 13.48]	42.9 %	0,33			
	Others	9	9	2	0.9	[0.12 - 4.86]	22.2 %	0,91			

Abbreviations: BMI=body mass index; ER=oestrogen receptor; PR=progesterone receptor; TNBC= triple negative breast cancer; str TILs= stromal tumor-infiltrating lymphocytes ; IT TILs= intratumoral-infiltrating lymphocytes ; NAC=neoadjuvant chemotherapy ; AC=anthracyclines ; pCR=Pathologic complete response

References

1. Wolff, A.C.; Hammond, M.E.H.; Schwartz, J.N.; Hagerty, K.L.; Allred, D.C.; Cote, R.J.; Dowsett, M.; Fitzgibbons, P.L.; Hanna, W.M.; Langer, A.; et al. American Society of Clinical Oncology/College of American Pathologists guideline recommendations for human epidermal growth factor receptor 2 testing in breast cancer. *J. Clin. Oncol.* 2007, *25*, 118–145, doi:10.1200/JCO.2006.09.2775.
2. WHO Classification of Tumours of the Breast. Fourth Edition - WHO - OMS - Available online: http://apps.who.int/bookorders/WHP/detart1.jsp?sesslan=1&codlan=1&codcol=70&codcch=4004 (accessed on Feb 9, 2018).
3. Symmans, W.F.; Peintinger, F.; Hatzis, C.; Rajan, R.; Kuerer, H.; Valero, V.; Assad, L.; Poniecka, A.; Hennessy, B.; Green, M.; et al. Measurement of residual breast cancer burden to predict survival after neoadjuvant chemotherapy. *J. Clin. Oncol. Off. J. Am. Soc. Clin. Oncol.* 2007, *25*, 4414–4422, doi:10.1200/JCO.2007.10.6823.
